# Alike but not the same: Psychological profiles of COVID-19 vaccine skeptics

**DOI:** 10.1177/20551029241248757

**Published:** 2024-04-25

**Authors:** Ursula Voss, Karin Schermelleh-Engel, Leana Hauser, Mira Holzmann, Diana Fichtner, Sonja Seifert, Ansgar Klimke, Sabine Windmann

**Affiliations:** 1 9173 Goethe University, Frankfurt, Germany; 2 27172 VITOS Hochtaunus Psychiatric Hospital, Friedrichsdorf, Germany; 3 3647 Groningen University, Groningen, Netherlands; 4 9170 Heinrich-Heine-Universität Düsseldorf, Dusseldorf, Germany

**Keywords:** conspiracy, COVID-19, latent profile analysis, pandemic, psychological profiles, scale construction, vaccine skepticism

## Abstract

One of the challenges of the SARS-CoV-2 pandemic was a widespread skepticism about vaccination. To elucidate the underlying mental and emotional predispositions, we examined a sample of 1428 participants using latent profile analysis (LPA) on selected personality trait variables, mental health status, and measures of irrational beliefs. LPA revealed five distinct profiles: two classes of non-skeptics and three of skeptics. The smaller non-skeptic class reported the highest rates of mental health problems, along with high levels of neuroticism, hostility, interpersonal sensitivity, and external locus of control. The larger non-skeptic class was psychologically well-balanced. Conversely, the skeptic groups shared strong distrust of COVID-19 vaccination but differed in emotional and mental profiles, leading to graded differences in endorsing extreme conspiracy beliefs. This suggests that vaccine skepticism is not solely a result of mental illness or emotional instability; rather extreme skepticism manifests as a nuanced, graded phenomenon contingent on personality traits and conspirational beliefs.

## Introduction

As of February 2024 (Worldometer, 2024), over 700 million people worldwide were reported to have been infected with the Severe Acute Respiratory Syndrome Coronavirus 2 (SARS-CoV-2) which causes Coronavirus Disease 2019 (COVID-19). While most of those infected have recovered, at least to a large extent, almost seven million people have died (WHO COVID-19 dashboard, 2024), not including estimates of underreporting (Böhning et al., 2020; Cumming-Bruce, 2021).

In total, it has taken the world more than 3 years to bring the virus largely under control. In the aftermath of the crisis, the most pressing questions are how we could have reduced its toll, and how well we are prepared for any future outbreaks. The question becomes particularly pertinent considering the multitude of infectious diseases expected to spread extensively with the changing global climate (Rocklöv et al., 2023; Semenza et al., 2022).

One of the major challenges of the SARS-CoV-2 pandemic was overcoming the widespread skepticism about COVID-19 vaccination (Aw et al., 2021; Sallam, 2021). Despite extensive information campaigns, vaccination rates remained suboptimal even when the vaccine was easily available. By the official conclusion of the pandemic, only 76% of the German population and 67% of the global population had been fully vaccinated (Holder, 2023). In light of the elevated mortality rates and substantial susceptibility to infection, refusing effective vaccination may seem irrational. With our study, we aimed to explore which distinct personality profiles and psychopathological tendencies might manifest themselves in notable skepticism.

Research has identified quite a number of potentially relevant sociodemographic and psychological characteristics of vaccination skeptics. While results on age and gender are inconclusive (Aw et al., 2021; Piltch-Loeb et al., 2022; Soares et al., 2021), disbelief in the severity of the disease seems to play a role, as does mistrust in governmental agencies and healthcare systems (Jamieson et al., 2021), lower education, and, more generally, a tendency toward conspiracy beliefs (Rossen et al., 2019). Social media provide a platform for spreading such theories (Garrett, 2020; Romer and Jamieson, 2020).

Other psychological variables identified as contributing to vaccine skepticism include political orientation, presumption of threat, mistrust in authorities, and the wish to be in control (Douglas et al., 2019; Fridman et al., 2021; Hagger and Hamilton, 2022; Jamieson et al., 2021; Jolley and Douglas, 2014). As for the libertarian desire for autonomy in decision-making (Rossen et al., 2019), research indicates that the perception of COVID-19 being beyond personal control (external locus of control) is associated with increased emotional distress, notably hostility (Šrol et al., 2022). Hostility, in turn, has been associated with high interpersonal sensitivity, characterizing an increased apprehension of threat and a tendency toward paranoid ideation (Bonab and Koohsar, 2011; Masillo et al., 2016; Mathes et al., 2019; Meisel et al., 2018). While data specifically on interpersonal sensitivity are not yet available, it seems plausible that hostility might be positively related to conspiracy beliefs and a willingness to engage in violent protests (Šrol et al., 2022).

Thus, for the most part, psychological trait variables of the low-functional and less agreeable kind appear to work together with context-specific psychological beliefs and attitudes in rendering individuals vulnerable to conspiracy theories. Several studies show an association between conspiratorial beliefs and certain personality factors, for example, high levels of openness to experience (Holm, 2009), low agreeableness (Galliford and Furnham, 2017), and high levels of neuroticism (Charlton, 2014). Regarding COVID-19 hesitancy, skeptics have been reported to have lower interoceptive awareness and cognitive empathy in the post-pandemic COVID-19 state (Vicario et al., 2024), trust in government agencies (Holford et al., 2023; Yuan et al., 2024). Interestingly, however, vaccine hesitancy in individuals with typically high scores on neuroticism, that is, psychiatric patients, appears to be rather low (Mazereel et al., 2021; Ren et al., 2021), pointing to the need for a more differentiated research approach. Controversial reports concern external and internal control beliefs, some reporting evidence for a stronger belief in fate and chance, others finding a higher internal locus of control (Murphy et al., 2021; Olagoke et al., 2021), suggesting that negative attitudes towards vaccination may not be a uniform issue. Presumably, not all vaccine skeptics share the same psychological profile (Fasce et al., 2023; Holford et al., 2023).

To summarize, it has been established that unvaccinated individuals are often prone to conspiracy theories (Holford et al., 2023; Milošević Đorđević et al., 2021; Romer and Jamieson, 2020; Sallam et al., 2021). However, most prior studies have focused only on a dichotomous comparison of the vaccinated and the unvaccinated (Edwards et al., 2021; El-Mohandes et al., 2021). Albeit necessary and interesting as a first step, these studies might inadvertently strengthen the notion of two steady, incompatible camps, with educated, functional, and agreeable vaccination proponents on the one hand and uneducated and psychologically unbalanced skeptics on the other (Šrol et al., 2022). In reality, however, it is not even clear whether and in how far temporally stable and domain-general personality traits as opposed to COVID-19-specific motives or beliefs, or a combination of the two, may underlie vaccination acceptance on the one hand and vaccination refusal on the other. More differential profiling may help to understand the major motivations behind vaccination skepticism (Fasce et al., 2023; Holford et al., 2023; Rossen et al., 2019).

Thus, the present study aimed to detect subgroups among groups of skeptics and non-skeptics based on COVID-19-specific attitudes and conspiratorial beliefs as well as psychological trait variables. For this purpose, the first aim was to develop and psychometrically evaluate a new scale, the COVID-19 Vaccine Attitudes and Beliefs Scale (VABS), measuring a broad range of reasons against COVID-19 vaccination. The second aim was to identify subgroups of skeptics and non-skeptics using a latent profile analysis (LPA) (Lazarsfeld and Henry, 1968) based on VABS, specific conspiratorial beliefs, and personality traits. The third aim was to investigate the subgroups’ characteristics in more detail with regard to possible emotional instability and their reasons for and against being vaccinated.

## Method

### Participants

Participants were recruited through flyers posted in general practitioners’ offices, pharmacies, and COVID-19 test centers in the German federal states of Hesse, Baden-Wuerttemberg, Bavaria, and Thuringia, as well as through announcements on social media (e.g.*, Instagram, Facebook, Twitter,* etc.) and the websites of Goethe University Frankfurt and the Psychiatric Hospital Vitos Hochtaunus. Five 30 Euro Amazon vouchers were raffled off among the participants willing to share their email addresses. Email addresses were stored independently of the survey answers by an algorithm provided by the software provider SoSciSurvey (Leitner, 2019).

Only participants who were at least 18 years of age, provided informed consent, and gave information on their current immunization status were included in the analysis. Five data sets had to be excluded because of a suspicious response pattern (the same response was given on practically all items). In total, data from 1248 participants (394 males, 843 females (76.6%), and 11 divers) were analyzed.

Participants were between 18 and 70+ years old. A total of 915 participants were vaccinated (73.3%) and 333 were unvaccinated (26.7%). Levels of education ranged from ≤ 9 years of schooling to a doctorate (for further details, see Supplemental Material, Table S1).

In addition to sociodemographic information, participants were required to disclose if they were suffering from a psychiatrically diagnosed mental health condition (yes/no). They were also asked to specify any diagnosed disorders (voluntary). Among the sample, 217 individuals (17.39%) reported having been diagnosed with a psychiatric illness, with 121 of those (55.76%) sharing their concrete diagnoses (see Table S11).

### Research procedures

Data were acquired online from July through October 2021. Informed consent was obtained from all participants included in the study. Data acquisition was anonymous. The following standardized measures were obtained.

**COVID-19 Vaccination Attitudes and Beliefs (VAB)** were addressed by 27 items answered on a Likert-type rating scale ranging from 1 = *do not agree at all* to 7 = *completely agree*. Item selection was conducted in agreement with best practices (e.g., Somma et al., 2022) as described in section “Statistical Analysis” and the Supplemental Material (pp. 3-9). About half of the items were taken from prior questionnaires (El-Mohandes et al., 2021; Jolley and Douglas, 2014; Piltch-Loeb et al., 2022; Shapiro et al., 2018), while others were derived from statements made either in interviews conducted with students and patients or on Internet forums and social media channels (e.g., “It is better to get sick from COVID then to get the vaccine”). Of the finally selected 14 items (see Table 1), items 3, 5, 6, 10, 13 have been adapted from Shapiro et al. (2016) while items 1, 2, 4, 7, 8, 11, 12 have been adapted from Jolley and Douglas (2014). The short scale showed excellent reliability (McDonald’s ω = 0.97).

**Table 1.** The COVID-19 Vaccination Attitudes and Beliefs Scale (COVID-19 VABS) with means (M), standard deviations (SD), and factor loadings (λ).

	Item content	*M*	*SD*	λ
1	COVID vaccines are often advertised for profit reasons.	3.129	0.062	0.850
2	Pharmaceutical companies cover up the dangers of COVID vaccines.	3.086	0.062	0.927
3	Data on the effectiveness of COVID vaccines are often fabricated.	2.660	0.056	0.908
4	I feel misinformed about the safety of COVID vaccines.	2.855	0.063	0.895
5	COVID vaccines are harmful to one’s health and this fact is being covered up.	2.545	0.060	0.929
6	COVID vaccines are harmless (inverted item).	3.851	0.055	0.735
7	The long-term consequences of COVID vaccines are still unexplored.	5.360	0.053	0.517
8	When it comes to COVID vaccines, I feel helpless.	2.772	0.062	0.811
9	It is better to get sick from COVID than to get the vaccine.	2.346	0.059	0.726
10	Vaccines will not stop the COVID pandemic.	2.973	0.063	0.828
11	I feel cheated, deceived, tricked by those responsible for the COVID vaccines (e.g., government, pharmaceutical companies, etc.).	2.708	0.066	0.927
12	I am unsure about the motives of those involved in the COVID vaccines (e.g., government, pharmaceutical companies, etc.).	3.051	0.066	0.879
13	Pharmaceutical companies, scientists and politicians are working together to cover up the dangers of COVID vaccines.	2.349	0.060	0.902
14	COVID vaccines are often contaminated.	2.253	0.048	0.795
	Reliability: ω = 0.972 (90% CI: 0.970, 0.974)			

Note. ω: McDonald’s omega.

**Extreme Conspiracy Beliefs** were measured by two items **“Tiny devices”** and **“Guinea pig” **which refer to descriptions of invasive physical manipulations believed by some extreme skeptics to be executed via COVID-19 vaccines. One of these items was adopted from postings on anti-vaxx social media channels which had attracted much press attention (e.g., Schoolov, 2021): “Getting the COVID vaccine turns me into a guinea pig for genetic manipulation”. The second item “Tiny devices are placed in COVID vaccines to track people’s movements” was taken from Jolley and Douglas (2014) (Study 1, item 7). Both items were provided alongside the same rating scale as the VABS items. The items were not added to the VABS because they significantly worsened the model fit and led to modification indices indicating that several error covariances should be freely estimated (see Supplemental material, p. 9). Therefore, the mean value of the two items was used as a measure of extreme conspiracy beliefs.

The **Internality, Powerful Others, and Chance Scale** (IPC) (Levenson, 1973, 1981) is one of the most widely used scales measuring general locus of control (Furnham and Steele, 1993). The IPC includes 24 items on three different subscales, one pertaining to internal locus of control, that is, the degree to which one attributes success and failure to one’s own efforts and abilities (subscale I_nternal_) and two subscales measuring external control (P_ower_ and C_hance_), that is, one’s conviction that life is determined mostly by chance or chaos (C_hance_). Sum scores are computed for each scale. Acceptable psychometric properties have been reported for all scales, with α = 0.81 for P_ower_, α = 0.83 for C_hance_, and α = 0.67 for I_nternal_ (Roddenberry and Renk, 2010). A seven-point Likert scale was applied (1 = *do not agree at all* to 7 = *completely agree*).

**Interpersonal sensitivity and hostility** were assessed with two subscales of the **Brief Symptom Inventory (BSI),** comprising 53 items measuring psychological distress and somatic comorbidities on nine symptom dimensions including interpersonal sensitivity and hostility. Respondents could indicate their intensity of distress during the past 7 days on a five-point Likert scale (1 = *does not at all apply* to 5 = *strongly applies*). Cronbach’s alpha ranges from 0.68 to 0.91 (Muthén and Muthén, 1998-2017). For the LPA, the five-point ratings were transformed into seven-point ratings.

**The Big Five Inventory-SOEP (BFI-S)** consists of 15 items covering five personality dimensions, of which we selected **openness to experience** and **neuroticism**. A seven-point response scale was used (1 = *not true at all* to 7 = *very true*). Cronbach’s alpha is relatively low with α = 0.60 for neuroticism and α = 0.63 for openness to experience. However, as the authors point out, with only three items per subscale, coefficients in this range are to be expected (Dehne and Schupp, 2007; Schupp and Gerlitz, 2008).

### Statistical analysis

SPSS version 28 was used for analyzing frequencies in contingency tables using likelihood-ratio χ^2^ tests as well as investigating mean difference tests between groups using independent *t*-tests. In case of variance heterogeneity, corrected test statistics were used.

For the development of the unidimensional 14-item VABS, exploratory factor analysis with Geomin rotation was performed first. As the results indicated that the scale was not unidimensional and that up to six factors no model fitted the data (Supplement, Table S4), we performed a confirmatory factor analysis applying the robust maximum likelihood estimator of the M*plus* program (Muthén and Muthén, 1998-2017). Based on similar item content (excluding items dealing with violent actions) and modification indices, the number of items was reduced stepwise from 27 to 14. A confirmatory factor analysis (CFA) was performed to test the model fit, and also to estimate the scale’s reliability via McDonald’s omega. Logistic regression with VABS as the predictor was used for the classification of vaccinated versus unvaccinated persons to pilot-test criterion validity of the VABS.

Latent profile analysis (LPA) (Lazarsfeld and Henry, 1968), a person-centered approach to latent variable analysis that belongs to the same family as cluster analysis and mixture modeling methods, was applied to identify latent subgroups within a sample based on patterns of responses to observed variables. Using again the MLR estimator of the M*plus* program, multiple models were fit with increasing numbers of profiles to identify the optimal number of latent profiles regarding eight psychological input variables, specifically neuroticism, external locus of control (chance and powerful others), hostility, interpersonal sensitivity, VABS, and extreme conspiracy beliefs. The final number of latent profiles was chosen based on theoretical plausibility and conceptual interpretability of the profiles as well as on statistical indices: Akaike information criterion (AIC) (Akaike, 1987), Bayesian information criterion (BIC) (Schwarz, 1978), sample size-adjusted BIC (SABIC) (Sclove, 1987), adjusted Lo-Mendell-Rubin likelihood-ratio test (aLRT) (Lo et al., 2001), bootstrap likelihood-ratio test (BLRT) (Nylund et al., 2007), and entropy (Celeux and Soromenho, 1996). For AIC, BIC, and SABIC, a larger drop between competing models suggests stronger support for the model with the lower values. For the comparison of nested models with increasing numbers of profiles, aLRT and BLRT values indicate that the given model with *k* profiles is significantly superior to the less parsimonious model with *k* - 1 profiles. Entropy values > .80 indicate a high level of separation between classes (Muthén and Muthén, 1998-2017). All these indices will be reported up to the accepted number of profiles.

Data are freely available at https://osf.io/q6puz/files/

### Compliance with ethical standards

The Ethics Committee of Goethe University Frankfurt grants a waiver to anonymous online surveys. The survey was conducted in accordance with the Helsinki Declaration as revised in 2013.

## Results

### COVID-19 vaccination attitudes and beliefs scale (COVID-19 VABS)

The CFA of the 14-item VABS showed that the single factor model fitted the data well (χ^2^(77) = 296.35, *p* < .01, RMSEA = 0.05, CFI = 0.98, SRMR = 0.02). Items of the final version of the COVID-19 VABS are listed in Table 1 (items are translated from German), age and gender distributions are given in the Supplemental Material (Figure S1 and Table S6).

As a first attempt toward validation, logistic regression was performed to predict vaccination status from the VABS scores. The model was statistically significant with χ^2^(1) = 718.39, *p* < .001, and explained 69% (Nagelkerke R^2^) of the variance in vaccination. Of *N* = 1144 valid cases, 89.4% were correctly classified compared to 74.5% in the null model, that is, the model with just the intercept included. These results provide preliminary evidence of the scale’s criterion validity.

### Latent profile analysis

**Figure 1.**
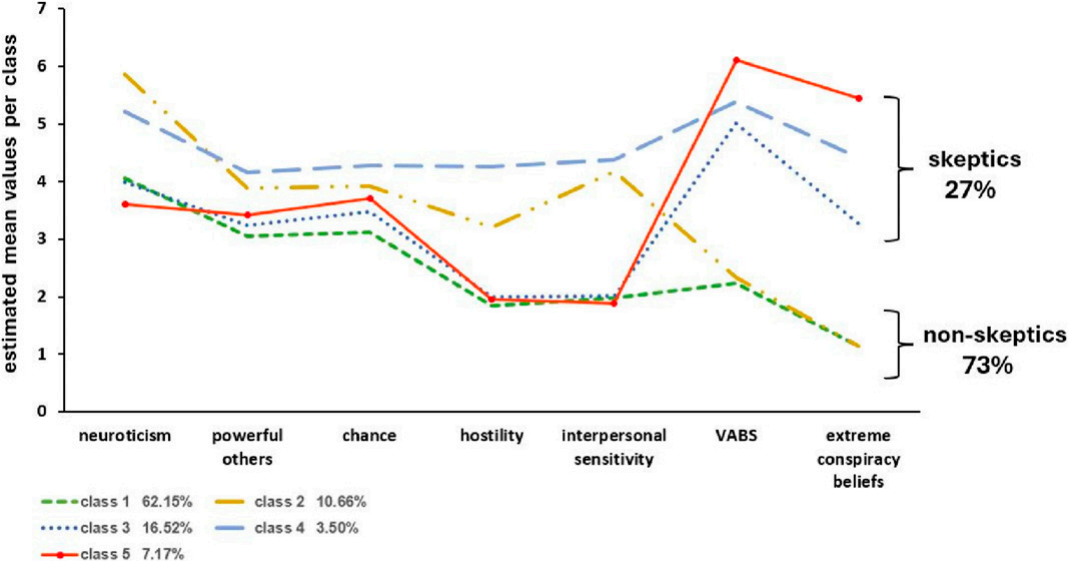
Latent profiles of vaccine non-skeptics and skeptics characterized by their patterns (estimated mean scores, individual scores ranging from 1 to 7) of personality traits, COVID-19-specific attitudes, and conspirational beliefs. *Note: N* = 1144. Final class proportions are based on the most likely latent class membership.

Table 2 presents model fit statistics for LPA models with increasing numbers of profiles. Between the models with 2 to five classes, increasingly lower values of log-likelihood values, AIC, BIC, and SABIC were observed as well as high entropy values for all models. As the model with five classes provides a significantly better fit to the data than the model with four classes (aLMRT: *p* = .001, BLRT: *p* < .001, entropy value = .935), and because of the theoretical interpretability and meaningfulness (see Figure 1), we decided to retain model 5 with five distinct profiles. Although the smallest class only contained 3.7% of the sample, this profile was retained because it adds a substantial new variable formation to the previous solution with four classes (Spurk et al., 2020).

**Table 2.** Model fit statistics for models with up to six latent profiles (*N* = 1144).

No. of profiles	LL	#FP	AIC	BIC	SABIC	Entropy	*p* (aLMRT)	*p* (BLRT)	Smallest class (%)
1	−11,306.20	14	22,640.40	22,711.00	22,666.53				
2	−10,262.70	22	20,569.39	20,680.32	20,610.44	0.972	0.000	0.000	24.3
3	−9828.98	30	19,717.96	19,869.22	19,773.93	0.916	0.065	0.000	11.8
4	−9529.54	38	19,135.09	19,326.69	19,205.99	0.928	0.004	0.000	9.0
5	−9305.98	46	18,703.97	18,935.91	18,789.80	0.935	0.001	0.000	3.7
6	−9128.72	54	18,365.44	18,637.72	18,466.20	0.907	0.758	0.000	7.3

Note. LL: log-likelihood; # FP: number of free parameters; AIC: Akaike information criteria; BIC: Bayesian information criteria; SABIC: sample-size-adjusted BIC; aLMRT: adjusted Lo-Mendell-Rubin likelihood-ratio test; BLRT: bootstrapped likelihood-ratio test.

**Figure 2.**
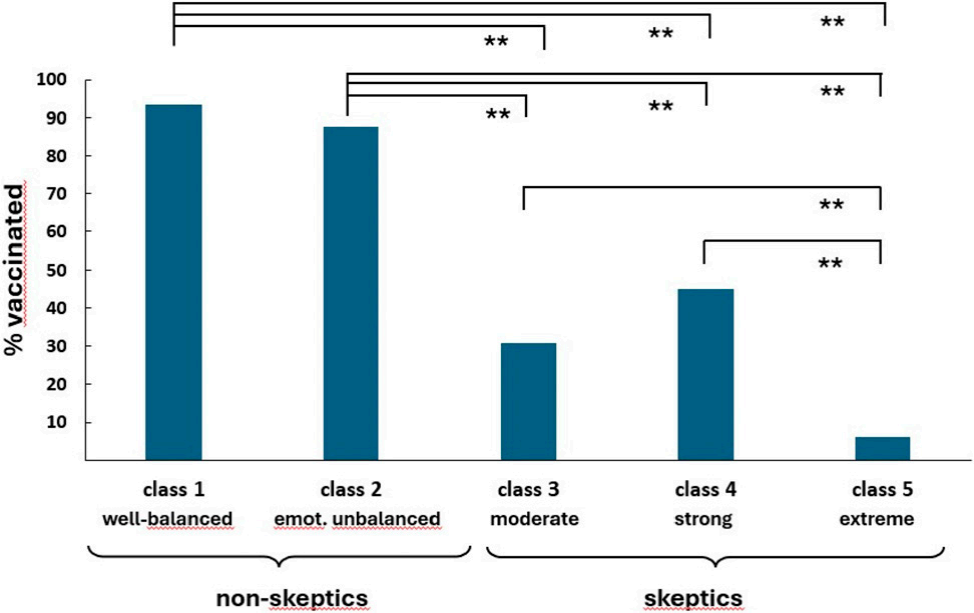
Percentage of vaccinated participants in two classes comprising vaccine non-skeptics (class 1 and 2) and three classes comprising vaccine skeptics (class 3, 4, 5). *Note: N* = 1144, **p < .01.

Differences between the five latent classes are largely due to differences in the VABS and the extreme conspiratorial beliefs. Based on the resulting profiles, we considered classes 1 and 2 with lower mean values on the VABS and extreme conspiracy beliefs as *vaccine non-skeptics* and those in classes 3, 4, and 5 with high values on these variables as *vaccine skeptics.* As shown in Figure 2, only classes 1 and 2 include high percentages of vaccinated participants. Results of significance tests for comparing mean scores of all measures used for the LPA across the five classes are presented in the Supplement (Table S12). Information on the age and gender of subjects in the five classes is given in the Supplemental Material (Figures S2 and S3).

#### COVID-19 vaccine non-skeptics

**Figure 3.**
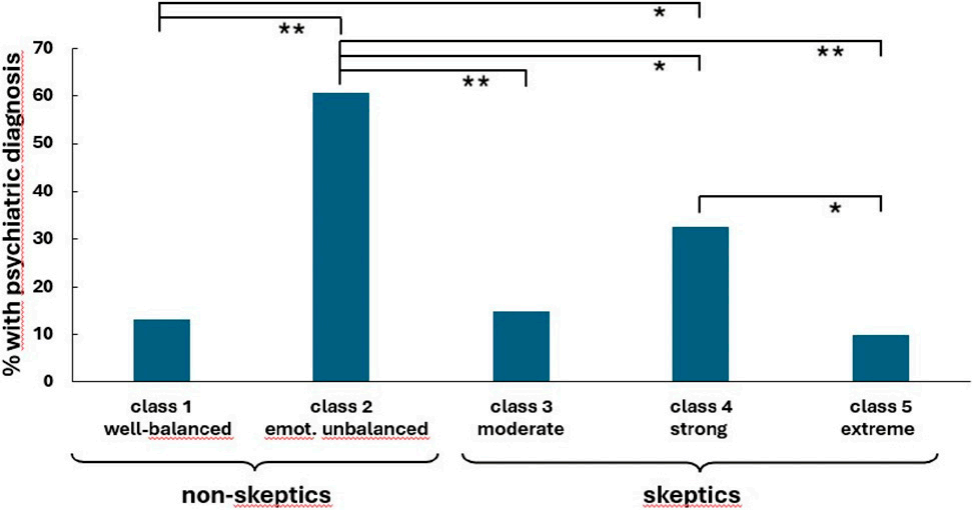
Percentage of participants in classes 1 to 5 who reported a diagnosed mental health condition. *Note: N* = 1144, **p < .01.

As shown in Figure 1, the majority of non-skeptics are in the first subgroup. They are low-profile on all personality trait variables and vaccine-specific measures (class 1: well-adjusted non-skeptics, 62.15% of all participants). The second subgroup, albeit also in favor of vaccination (class 2: emotionally unbalanced non-skeptics, 10.66%), is characterized by high mean scores on neuroticism, hostility, and interpersonal sensitivity as well as external locus of control measures. Most participants of this class (60.7%) reported to be suffering from a diagnosed mental health problem that may relate to their distinct personality trait profiles (see Figure 3).

#### COVID-19 vaccine skeptics

Regarding the three subgroups of vaccine skeptics, the largest group (class 3, *moderate skeptics*: 16.5%) appears to be rather well-balanced regarding personality traits; yet, this class shows strong distrustful beliefs about COVID-19 vaccination as measured by the VABS (see Figure 1) while scoring moderately on extreme beliefs. A second subgroup (class 4, *strong skeptics*) makes up only 3.5% of all participants. This group is characterized by high values on all personality measures in addition to the vaccination-specific measures. A quite large number of participants in this class (32.5%) reported (unspecified) mental health problems that may relate to their distinct personality trait profiles (see Figure 3). Interestingly, most individuals in class 1 willingly disclosed their diagnosis (93.6%). Conversely, not one of those grouped as skeptics was willing to share their specific diagnosis (see Table S11).

The third subgroup (class 5, *extreme skeptics*: 7.1%) shows a low profile in the trait measures, similar to class 3, but displays higher mean scores than any other subgroup on the VABS, and the highest agreement with extreme conspiratorial beliefs.

*In summary,* all three subgroups of vaccine skeptics endorsed the extreme beliefs describing inhumane invasive procedures (averaged across the two items “Guinea Pigs” and “Tiny Devices”). A comparison of the merged skeptics classes with the merged non-skeptics classes with regard to the extreme conspiratory beliefs revealed a significant difference, *t* (324.15) = −41.89, *p* < .001, with higher scores for the skeptics (*M* = 3.98, *SD* = 1.18, *N* = 832) compared to the non-skeptics (*M* = 1.14 *SD* = 0.31, *N* = 309). Their absolute agreement to this measure was, on average, almost four times higher than that of the non-skeptics. Generally, extreme conspirational beliefs greatly distinguished the three subgroups of skeptics from the two subgroups of non-skeptics, but it also showed some graded differences between the three classes of skeptics.

### Reasons for and against vaccination

**Figure 4.**
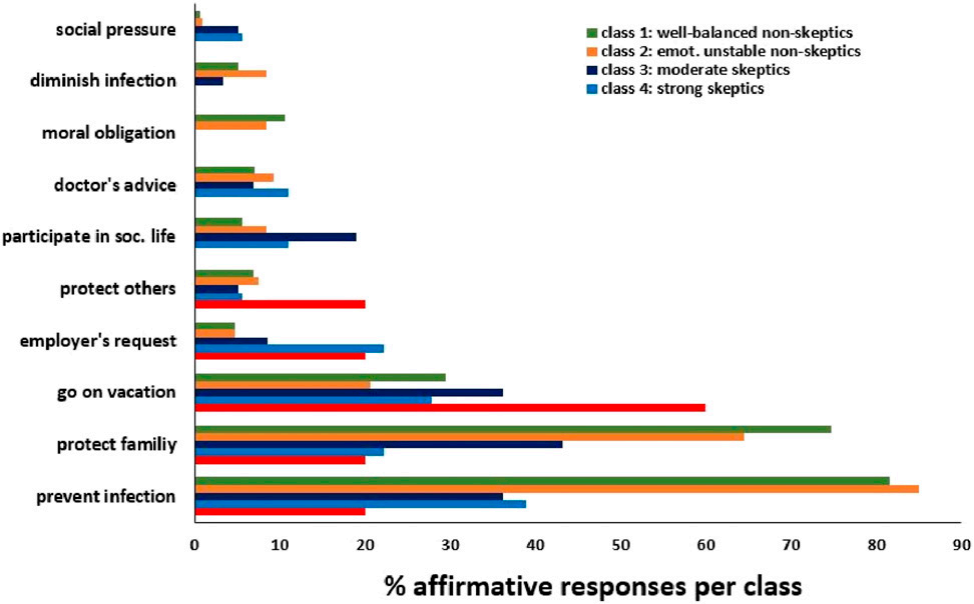
Reasons of vaccinated individuals that led to vaccination. Multiple answers were allowed (*N* = 915).

Next, we were interested in reasons for and against vaccination. Figure 4 shows the reasons that led to vaccination in non-skeptics and skeptics. According to the much smaller percentage of vaccinated individuals in the three skeptics groups, affirmative responses are less frequent in these groups. As can be seen from Figure 4, for vaccinated individuals in the non-skeptic groups as well as for vaccinated individuals in the skeptic groups, the most common reason for vaccination was infection prevention, followed by the wish to protect family members and the desire to go on vacation. By contrast, social pressure, diminishing an infection, protection of unacquainted others, or moral obligations towards society were rarely mentioned, and if so, mostly from the two groups of non-skeptics.

Figure 4 depicts the responses of vaccinated individuals from the two non-skeptics groups and the three skeptics groups. The most frequent reasons leading to vaccination in the non-skeptic groups were to prevent an infection and to protect family members. Being allowed to go on vacation was a strong motive for the skeptics groups, especially for extreme skeptics. The items “social pressure”, the wish to “mitigate an infection”, or “moral obligation towards society” were only seldom affirmed by either group.

**Figure 5.**
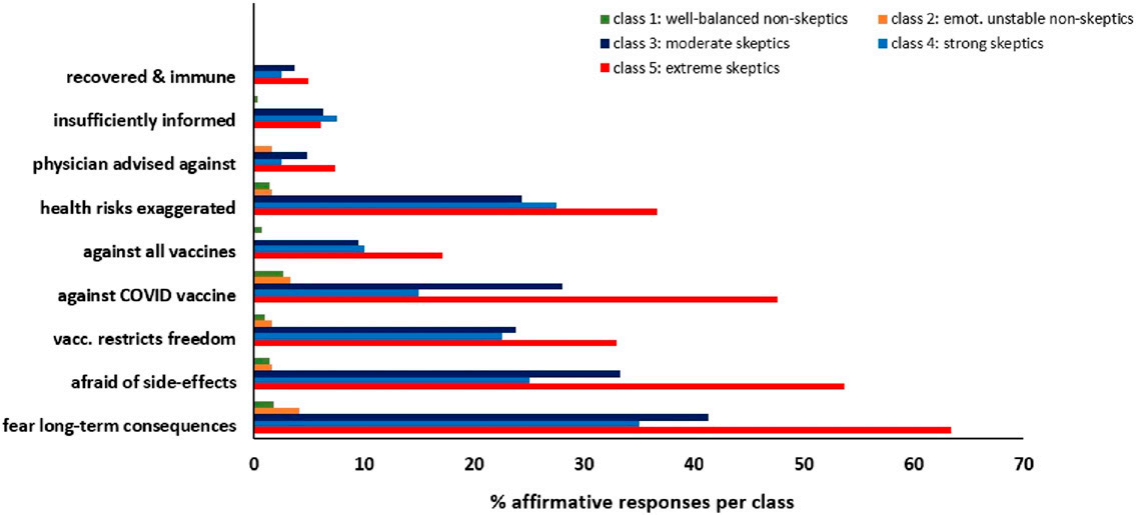
Reasons of unvaccinated individuals that led to vaccination refusal. Multiple answers were allowed (*N* = 259).

Figure 5 shows the responses of unvaccinated individuals from the three skeptics groups and the two non-skeptics groups. The most frequent concern leading to a refusal of vaccination in all five groups was the fear of long-term consequences. Among the three groups of skeptics, this concern was expressed more frequently by moderate and strong skeptics compared to extreme skeptics. Moderate and strong skeptics also more frequently reported to believe that health risks of the COVID-19 disease were exaggerated. By contrast, extreme skeptics most frequently affirmed the statements that they were against all vaccines and that vaccination restricts their freedom. Only a few participants felt insufficiently informed. The few ratings for all items by the two groups of non-skeptics reflect the low rate of unvaccinated individuals in these groups.

## Discussion

As a more differentiated approach than usually taken, we used the newly developed VABS, the average of two extreme conspiracy items, and measures of relevant personality traits to identify distinct psychological profiles among vaccine non-skeptics and vaccine skeptics. Specifically, we asked whether vaccine skeptics might be more prone to mental and emotional instability than vaccine non-skeptics.

Our results suggest, first and foremost, that vaccine skepticism depends more on conspiratorial beliefs about vaccination than on psychological trait measures. Specifically, latent profile analysis identified a total of five classes, of which two mostly entail vaccine non-skeptics and the remaining three vaccine skeptics. Both subgroups of *non-skeptics* scored low on the VABS and the extreme conspiracy beliefs measured by the items “Tiny Devices” and “Guinea Pig”. The larger one of these two classes (class 1) was entirely neutral regarding psychiatric symptoms, hostility, locus of control, or interpersonal sensitivity. The smaller subgroup, however, which we labeled “emotionally unbalanced” (class 2), was characterized by high levels of neuroticism, increased hostility, interpersonal sensitivity, and an external locus of control. Almost two-thirds of individuals within this subgroup disclosed a documented psychiatric diagnosis. However, the fact that this subgroup nevertheless supports vaccination and scores low on the VABS, let alone the extreme conspiracy items, shows that emotional instability is not necessarily linked to ideological thinking, as suggested elsewhere (Charlton, 2014). Possibly, this group of non-skeptics may be generally more compliant toward medical interventions because they have more prior experience with and higher trust in medical practices.

Within the cohort of vaccine *skeptics*, the largest subgroup (class 3: *moderate skeptics*) exhibits trait measures comparable to class 1 non-skeptics, reflecting a well-balanced psychological profile. Importantly, however, this subgroup differs from both subgroups of non-skeptics regarding their higher VABS scores and their higher conspiracy beliefs.

The groups of strong and extreme skeptics (class 4 and 5) appear more poorly educated than the two groups of non-skeptics, and they are characterized by high scores throughout. Their scores on neuroticism, hostility, interpersonal sensitivity, and external locus of control are even higher than those of the non-skeptical class 2, and VABS and extreme conspiracy beliefs are higher than those of the skeptical class 3. These individuals are to be considered hostile, and they seem to exhibit a high degree of interpersonal sensitivity in the sense of heightened apprehension of threat and danger to self. Since the group is very small, which makes their reliability somewhat uncertain, future studies need to confirm these interpretations (Marsh et al., 2009; Masyn, 2013).

Finally, the most striking subgroup is class 5 (*extreme skeptics*). This group is also relatively small (7.17%) but holds extreme views regarding vaccination, in particular against COVID-19. While 14.6% of this group has a low level of education, 64.7% are well educated, holding at least a high school degree, 14.6% being currently enrolled as university students, and 22.0% even holding a university degree (see Supplemental Material, Figure S4). Trait-wise, they appear to be balanced and stable, but they show strong distrust against COVID-19 vaccination as indicated by high VABS scores and extreme conspiracy beliefs. The latter suggests that they endorse what Holford et al. (2023) have identified as “Conspiracist Ideation”, an extreme mindset that goes beyond distrust and fear by believing in “alternative causes” without objective evidence. This strong irrational conviction might make them oblivious to scientific arguments about the benefits of inoculation (Lampl, 2022). The same may be true for classes 4 and 3, although to a lesser extent.

In summary, using our measures, we found a clear-cut difference between skeptics and non-skeptics, unlike Rossen et al. (2019) who have described an additional group of “fence-sitters”. Interestingly, however, the most pronounced consensus among the three groups of skeptics, aside from the apprehension expressed in the VABS, is not found in their trait profiles, but in their agreement with the two extremely non-scientific conspiracy statements. In absolute terms, these items were almost exclusively endorsed by participants in the three subgroups of skeptics, which calls for closer semantic analysis. Unlike the VABS, the items conspicuously allude to dehumanizing medical procedures involving a profound alteration of one’s human identity, like feeling genetically manipulated like a guinea pig (Driedger et al., 2023; MacEwan et al., 2023). Naturally, individuals harboring suspicions that vaccination comprises their integrity and personal autonomy in this fundamental manner will reject the procedure without further consideration. However, according to our results, such extreme beliefs do not seem to be based on any abnormal trait characteristics, as only the smallest subgroup, class 2, but not the other two subgroups, classes 3 and 5, show such deviations.

In terms of the general implications of our study for the assessment of vaccine skepticism, the utility of the newly designed VABS may extend beyond investigations of the COVID-19 pandemic in future research. The selected items can be adapted to assess and evaluate vaccination hesitancy related to other virus infections (e.g., measles, polio, monkeypox, dengue). If the VABS proves transferable to the acceptance of other vaccines, it could serve as a valuable tool during the early stages of an evolving epidemic to identify (groups of) individuals who require special interventions such as reassessment (Jin et al., 2021) and/or inoculation (Van der Linden et al., 2021), even before attitudinal barriers are erected. Conversely, if the scale is not transferable, it will be instructive to delineate COVID-19-specific items from those with broader relevance. Items demonstrating specificity to COVID-19 could indicate unique intervention requirements, while context-insensitive ones may guide the development of generalizable intervention procedures. According to our results, individuals classified as most skeptical perceive vaccination as an identity-altering procedure that poses a threat to free will and self-determination. This belief might be particularly relevant for mRNA agents whose mechanism of action is complex and difficult to understand for laypersons.

Of course, our study is subject to certain limitations. First, although our recruitment strategy in GP offices, psychiatries, test centers, and via social media allowed us to sample a range of personalities, including those who had experienced or were currently experiencing health problems, it may not have resulted in a sample that is representative of the German adult population. Furthermore, we may not be able to generalize our findings to populations in other countries, although similar results regarding hostility and the association of antivaccination with conspiracy beliefs have been reported previously (Šrol et al., 2022).

Another limitation pertains to the use of the VABS, which has not undergone validation with independent samples as of yet. However, given the favorable psychometric properties observed for the VABS and its efficacy in distinguishing between vaccinated and unvaccinated individuals, we express confidence in its potential usefulness for further research.

In summary, our findings provide novel insights regarding skeptics and non-skeptics of COVID-19 vaccination. The LPA has successfully unveiled the latent psychological make-up of COVID-19 vaccine non-skeptics and skeptics. Attitudinal factors identified via the VABS, in conjunction with personality traits, delineate distinct profiles that may warrant differential intervention strategies. By contrast, demographic and psychological trait measures alone proved insufficient for accurately predicting skepticism toward COVID-19 vaccination. Except for the small class 4 (3.7% of the sample) in which hostile personality traits coincided with specific and extreme attitudes against COVID-19 vaccination, the remaining skeptics (23.2% of the sample) may benefit from interventions tailored to rectify their conspiracist ideation regarding the intentions behind COVID-19 vaccination.

## Supplemental Material

Supplemental Material - Alike but not the same: Psychological profiles of COVID-19 vaccine skeptics Supplemental Material for Alike but not the same: Psychological profiles of COVID-19 vaccine skeptics by Ursula Voss, Karin Schermelleh-Engel, Leana Hauser, Mira Holzmann, Diana Fichtner, Sonja Seifert, Ansgar Klimke and Sabine Windmann in Health Psychology Open

Supplemental Material - Alike but not the same: Psychological profiles of COVID-19 vaccine skeptics Supplemental Material for Alike but not the same: Psychological profiles of COVID-19 vaccine skeptics by Ursula Voss, Karin Schermelleh-Engel, Leana Hauser, Mira Holzmann, Diana Fichtner, Sonja Seifert, Ansgar Klimke and Sabine Windmann in Health Psychology Open

Supplemental Material - Alike but not the same: Psychological profiles of COVID-19 vaccine skeptics Supplemental Material for Alike but not the same: Psychological profiles of COVID-19 vaccine skeptics by Ursula Voss, Karin Schermelleh-Engel, Leana Hauser, Mira Holzmann, Diana Fichtner, Sonja Seifert, Ansgar Klimke and Sabine Windmann in Health Psychology Open

## Data Availability

Data are freely available at https://osf.io/q6puz/files/.
