# Supplementary material for: Alike but not the same: Psychological profiles of COVID-19 vaccine skeptics
Source: Health Psychol Open. 2024 Apr 25;11:20551029241248757. doi: 10.1177/20551029241248757 (PMC11047032; doi:10.1177/20551029241248757)

Supporting Information for:

Alike but not the Same: Psychological Profiles of COVID-19 Vaccine Skeptics

Ursula Voss, Karin Schermelleh-Engel, Leana Hauser, Mira Holzmann, Diana Fichtner, Sonja Seifert, Ansgar Klimke, & Sabine Windmann

Overview

Descriptives 2
Table S1 2
Exploratory Factor Analysis 3
Table S2 *Item pool for VABS* 3
Mplus Input File for EFA 4
Table S3 *Sample statistics* 5
Table S4 *Summary of model fit information* 6
Table S5 *Factor loadings of the three-factor solution with Geomin rotated factors* 6
Confirmatory Factor Analysis 7
Item Selection and Model Evaluation 7
Mplus Input File for CFA + Calculation of Omega 8
Extreme Conspiracy Beliefs 9
Differences in VABS between age groups and gender 10
Figure S1 *VABS mean scores by age groups* 10
Table S6 *VABS mean scores for gender groups* 11
Latent Profile Analysis (LPA) 11
Table S7 *Correlation Matrix of Vaccination Status and Measures used for LPA* 11
Mplus Input File for LPA 12
Table S8 *LPA output: loglikelihood (LL) values* 13
Table S9 *Average latent class probabilities for most likely latent class membership (row) by latent class (column)* 14
Table S10 *Final class counts and proportions for the latent classes based on their most likely class membership* 14
Table S11 *Distribution of mental health problems across latent classes* 14
Table S12 *Significance Tests for Class Comparisons per Construct.* 15
Figure S2 *Percentages of class membership for each age group* 17
Figure S3 *Percentages of class membership for each gender group* 18
Figure S4 *Percentage of level of education by latent class membership* 19

Descriptives

Kommentiert [UV1]: Diese ganze Tabelle ist jetzt neu berechnet

Table S1

Descriptive statistics for vaccination status

	Vaccination Status			
	Vaccinated Twice *N* (%)	Vaccinated Once *N* (%)	Unvaccinated *N* (%)	Total *N* (%)
Sample size	858 (68.8)	57 (4.6)	333(26.7)	1248 (100)
Age				
18 – 29	409 (73.3)	40 (7.2)	109 (19.5)	558 (44.7)
30 – 39	171 (66.3)	6 (2.3)	81 (31.4)	258 (20.7)
40 – 49	98 (59.4)	5 (3.0)	62 (37.6)	165 (13.2)
50 – 59	118 (67.0)	2 (1.1)	56 (31.8)	176 (14.1)
60 – 69	47 (64.4)	4 (5.5)	22 (30.1)	73 (5.8)
70 plus	15 (83.3)	--	3 (16.7)	18 (1.4)
Gender				
Female	579 (68.7)	39 (4.6)	225 (26.7)	843 (67.5)
Male	270 (68.5)	18 (4.6)	106 (26.9)	394 (31.6)
Divers	9 (81.8)	--	2 (18.2)	11 (.9)
Highest level of education				
≤ 9 years of school	6 (17.6)	2 (5.9)	26 (76.5)	34 (2.7)
Secondary school	55 (46.6)	7 (5.9)	56 (47.5)	118 (9.5)
Trade school	70 (50.0)	4 (2.9)	66 (47.1)	140 (11.2)
High school	119 (68.4)	7 (4.0)	48 (27.6)	174 (13.9)
Undergraduate	190 (75.7)	18 (7.2)	43(17.1)	251 (20.1)
Bachelor's degree	173 (78.3)	8 (3.6)	40 (18.1)	221 (17.7)
Master's degree	210 (78.1)	11 (4.1)	48 (17.8)	269 (21.6)
Doctorate	35 (87.5)	--	5 (12.5)	40 (3.2)
Physical health problems	191 (67.7)	11 (3.9)	80 (28.4)	282 (22.6)
Mental health problems	174 (74.4)	11 (4.7)	49 (20.9)	234 (18.8)

Note. Due to a few missing values (max. 8%), the sum of the percentages does not always equal 100%.

The percentages of vaccinated and unvaccinated persons were about equally distributed across men and women.

Exploratory Factor Analysis

The COVID-19 VABS was developed and psychometrically evaluated out of a pool of 27 items (see Table S2 for item wordings and Table S3 for sample statistics). To determine the number of factors in the data set, an exploratory factor analysis was conducted with increasing number of factors (see Table S4). Factor loadings on the first factor were almost always higher than loadings on the second and the third factor (see Table S5).

Table S2

Item pool for VABS

No.	Item wording
1	Data on COVID vaccine safety is often falsified.
2	COVID vaccines are often advertised for profit reasons.
3	Pharmaceutical companies cover up the dangers of COVID vaccines.
4	I feel misinformed about the effectiveness of COVID vaccines.
5	Data on the effectiveness of COVID vaccines are often falsified.
6	I feel misinformed about the safety of COVID vaccines.
7	The government is trying to cover up a link between COVID vaccines and cancer.
8	COVID vaccines are harmful to health and this fact is being covered up.
9r	COVID vaccines are harmless, recoded (inverted item).
10	The long-term consequences of COVID vaccines are still unexplored.
11	When it comes to COVID vaccines, I feel helpless.
12	It is better to get sick from COVID than to get the vaccine.
13	Vaccines will not stop the COVID pandemic.
14	I feel cheated, deceived, duped by those responsible for the COVID vaccines (e.g., government, pharmaceutical companies, etc.).
15	I am apprehensive about the potential side effects from the COVID vaccine.
16	I feel uncertain about the safety of the COVID vaccine.
17	The side effects of COVID vaccines are unpredictable.
18	COVID vaccines cause allergies.
19	I am unsure about the motives of those involved in the COVID vaccines (e.g., government, pharmaceutical companies, etc.).
20	COVID vaccines cause COVID.
21	I distrust those who are involved in the COVID vaccines (e.g., government, pharmaceutical companies, etc.).
22	I am disappointed with those involved in the COVID vaccines (e.g., government, pharmaceutical companies, etc.).
23	COVID vaccines allow governments to track and control people.
24	Pharmaceutical companies, scientists and politicians are working together to cover up the dangers of COVID vaccines.
25	COVID vaccines are often contaminated.
26	Tiny devices are placed in COVID vaccines to track people's movements.
27	If I get the COVID vaccine, I'm a guinea pig for genetic manipulation.

Exploratory Factor Analysis using Mplus, MLR estimator and Goemin rotation.

Mplus Input File for EFA

```
TITLE:      EFA 27 Items, Geomin Rotation

Data:       File = VABS.dat;
            !NOBSERVATIONS = 1248;

Variable:   Names =
            V1 V2 V3 V4 V5 V6 V7 V8 V9r V10 !V9 reverse formulated
            V11 V12 V13 V14 V15 V16 V17 V18 V19 V20
            V21 V22 V23 V24 V25 V26 V27;

            MISSING = ALL(-99);

Analysis:   ! Increasing number of factors from 1 to 6
            Type = EFA 1 6;
            ! Robust ML estimator
             Estimator = MLR;
            ! Rotation is Geomin
             Rotation = Geomin;

OUTPUT:     SAMPSTAT; ! Print sample statistics in output
```

Output EFA

Table S3

Sample statistics

Variable	N	Mean	Variance	Skewness	Kurtosis
V1	1147	2.770	3.508	0.913	-0.330
V2	1146	3.231	4.061	0.581	-0.953
V3	1146	3.200	4.006	0.658	-0.858
V4	1145	2.895	4.106	0.861	-0.624
V5	1146	2.757	3.384	0.957	-0.176
V6	1145	2.935	4.055	0.830	-0.656
V7	1145	2.233	2.710	1.250	0.689
V8	1145	2.653	3.716	0.972	-0.307
V9	1145	3.921	3.056	0.385	-0.952
V10	1145	5.392	2.736	-0.976	0.107
V11	1145	2.866	4.004	0.780	-0.725
V12	1145	2.465	3.746	1.113	-0.057
V13	1146	3.076	4.156	0.675	-0.894
V14	1145	2.815	4.621	0.868	-0.744
V15	1145	3.740	3.889	0.238	-1.207
V16	1145	3.418	4.156	0.514	-1.102
V17	1145	4.096	3.668	0.105	-1.250
V18	1145	2.966	2.473	0.388	-0.560
V19	1145	3.131	4.493	0.638	-1.024
V20	1145	1.957	2.310	1.648	1.982
V21	1144	3.152	4.361	0.633	-0.983
V22	1144	3.312	4.417	0.461	-1.155
V23	1144	1.788	2.184	1.949	2.905
V24	1144	2.454	3.800	1.129	-0.086
V25	1145	2.329	2.504	1.179	0.673
V26	1144	1.483	1.216	2.536	6.278
V27	1145	2.346	3.975	1.264	0.136

As the descriptive statistics show, skewness and kurtosis values of the variables indicate that the variables are nonnormally distributed. However, the deviation from the normal distribution is not too large, so that the robust maximum likelihood estimator was used for the analyses.

Table S4

Summary of model fit information

Model	Model Parameters	Chi-Square	Degrees of Freedom	P-Value
1-factor	81	2991.092	324	0.0000
2-factor	107	1829.025	298	0.0000
3-factor	132	1377.365	273	0.0000
4-factor	156	853.227	249	0.0000
5-factor	179	658.341	226	0.0000
6-factor	201	526.489	204	0.0000

Even the six-factor solution did not provide a good model fit. Below we show the factor loading matrix for three factors of the Mplus output.

Table S5

Factor loadings of the three-factor solution with Geomin rotated factors

**Item No.**	**Factor 1**	**Factor 2**	**Factor 3**
V1	0.870*	0.037	-0.130*
V2	0.920*	-0.079*	-0.051
V3	0.966*	-0.040	-0.045
V4	0.928*	-0.071	-0.006
V5	0.920*	0.029	-0.110*
V6	0.955*	-0.088	0.027
V7	0.659*	0.264*	-0.053
V8	0.866*	0.094*	-0.001
V9R	0.759*	-0.056	0.069*
V10	0.530*	-0.094*	0.255*
V11	0.754*	0.001	0.237*
V12	0.618*	0.150*	0.053
V13	0.787*	0.059	0.019
V14	0.894*	0.042	0.022
V15	0.676*	0.007	0.448*
V16	0.754*	0.011	0.403*
V17	0.738*	-0.009	0.274*
V18	0.584*	0.191*	0.057
V19	0.882*	-0.027	0.095*
V20	0.498*	0.403*	-0.067
V21	0.889*	0.008	-0.003
V22	0.809*	-0.009	0.029
V23	0.218*	0.693*	0.008
V24	0.746*	0.252*	-0.022
V25	0.597*	0.314*	0.003
V26	0.013	0.874*	0.058
V27	0.691*	0.307*	0.006

* $p < .05$

Almost all items load onto the first factor, while a clearly defined second factor does not seem to exist, apart from V26, the “Tiny Devices” item which was assessed separately anyway.

We additionally performed a principal component analysis followed by a parallel analysis in order to determine the number of factors. Principal component analysis revealed that there were only two eigenvalues > 1 (17.381, 1.104, 0.694, …), but parallel analysis (1.276, 1.236, 1.204, …) suggested keeping only one factor. We, therefore, decided to keep only one factor.

Confirmatory Factor Analysis

Item Selection and Model Evaluation

As the EFA did not result in clearly separable factors, a single factor CFA using 27 items was performed using the robust maximum likelihood method (MLR) of the M*plus* program, version 8.4 (Muthén & Muthén, 1998–2017). This estimator uses full information maximum likelihood (FIML) for handling missing data and takes non-normality of the data into account. Model fit was evaluated by the Yuan-Bentler (YB)-corrected χ^2-test (Yuan & Bentler, 2000) and its associated *p*-value, as well as some descriptive fit indices, i.e., root mean square error of approximation (RMSEA), comparative fit index (CFI), and standardized root mean square residual (SRMR). A good model fit is indicated by a nonsignificant YB-χ^2 (p > .05), RMSEA χ^2 < .05, CFI χ^2 > .97, and SRMR < .05 (Hu & Bentler, 1999; Schermelleh-Engel, Moosbrugger & Müller, 2003).

We received an unsatisfactory model fit with YB-χ^2(324) = 2991.092 ($p < .01$), RMSEA = .085 (90% CI: .082, .0848), CFI = .893, and SRMR = .040. Modification indices suggested relaxing several constraints related to correlated errors, but no indication was given that more than one factor should be used. Based on similar item content (excluding items about violent actions) and modification indices, the number of items was reduced stepwise to 14 (see input file below). This model now provided a good model fit with YB-χ^2(77) = 296.35 ($p < .01$), RMSEA = .050 (90% CI: .044; .056), CFI = .980, and SRMR = .016, establishing the factorial validity of the COVID-19 VABS. Factor loadings ranged between .517 and .929.

Mplus Input File for CFA + Calculation of Omega

```
TITLE:      CFA VABS 14 Items, 1 Factor, McDonald's Omega, CI

Data:       File = VABS14.dat;
            !NOBSERVATIONS = 1248;

Variable:   Names =
            V1 V2 V3 V4 V5 V6 V7 V8 V9r V10 !V9 reverse formulated
            V11 V12 V13 V14 V15 V16 V17 V18 V19 V20
            V21 V22 V23 V24 V25 V26 V27;

            USEVARIABLES =
            V2 V3 V5 V6 V8 V9r V10
            V11 V12 V13 V14 V19 V24 V25;

            MISSING = ALL(-99);

Analysis:   Estimator = MLR;

Model:      ! Factor loadings
            ! Labels, i.e., (p1), are needed for McDonald's omega
            Factor BY
            V2* (p1)
            V3-V25 (p2)-(p14);

            ! Latent variance fixed to zero for identification
             Factor@1;

            ! Error variances
            V2-V25 (e1)-(e14);

      ! If the model fits the data, omega can be calculated
      ! Model Constraint is needed for defining and estimating new parameters
      MODEL CONSTRAINT:

       NEW(True); ! True-Score Variance
      True = ((p1+p2+p3+p4+p5+p6+p7+p8+p9+p10+p11+p12+p13+p14)**2)*1.0;

      NEW(Error); ! Error Variance
       Error = (e1+e2+e3+e4+e5+e6+e7+e8+e9+e10+e11+e12+e13+e14);

      !Calculation of OMEGA
      NEW(OMEGA);  ! True-Score Variance/ Total Variance
      OMEGA = (True)/(True + Error);

      ! Asymmetric Confidence Interval
      ! see Raykov, T. (2002). Analytic estimation of standard error and
```

```
! confidence interval for scale reliability.
! Multivariate Behavioral Research, 37, 89–103.
 NEW(REL, SE, L, SEL, CI_L_LO, CI_L_UP, CI_R_LO, CI_R_UP);

REL = 0.972;                !Omega from first calculation
SE  = 0.001;                !SE taken from first calculation

L = LOG(REL/(1-REL));       !Logit transformation
SEL = SE/(REL*(1-Rel));  !SE of logit

    CI_L_LO = L - 1.96*SEL;     !CI-Low of logit
    CI_L_UP = L + 1.96*SEL;     !CI-Up of logit

    CI_R_LO = 1/(1+EXP(-CI_L_LO));  !CI-Low of Rel
    CI_R_UP = 1/(1+EXP(-CI_L_UP));  !CI-Up of Rel
```

OUTPUT: STDYX;

Item means, standard deviations, and factor loadings are listed in Table 1 in the manuscript.

Extreme Conspiracy Beliefs

Two items (V26 and V27) not loading on the common VABS factor were nonetheless retained in the subsequent analyses as single items because of their high indication of conspiracy theory. These items had circulated in anti-vaxx social media channels among extreme conspiracists, i.e., “Tiny devices are placed in corona vaccines to track people's movements” and “Getting the COVID vaccine turns me into a guinea pig for genetic manipulation”. Adding these two items to the VABS resulted in a worsened model fit, YB-$\chi^2(104) = 656.90$, $p < .01$, BIC = 55258.83 compared to the model fit of the 14-item VABS with YB-$\chi^2(77) = 296.350$, $p < .01$, BIC = 49368.13. Additionally, this analysis led to 15 modification indices > 10 related solely to items 26 and 27, indicating that several error covariances should be freely estimated. Therefore, these items were used separately as a measure of extreme conspiracy beliefs together with the VABS in the latent profile analysis.

Differences in VABS between age groups and gender

Age groups

Analysis of variance yielded a significant effect for age [$F(5, 1138) = 13.90$, $p < 0.01$]. Post hoc analyses using the Scheffé test indicated that the mean VABS scores were significantly higher for the middle-aged groups aged 40 – 49 years ($M = 3.78$, $SD = 1.89$) and 50 – 59 years ($M = 3.49$, $SD = 1.78$) compared to the groups of 18 – 29 years ($M = 2.71$, $SD = 1.36$) and 30 – 39 years ($M = 3.12$, $SD = 1.63$), but no significant differences were revealed of these groups with the older age groups 60 – 69 ($M = 3.34$, $SD = 1.93$) and 70+ ($M = 3.04$, $SD = 1.42$) (see Figure S1).

Figure S1

*VABS mean scores by age groups and standard errors. Item responses range from 1 to 7 (**p < .01)*

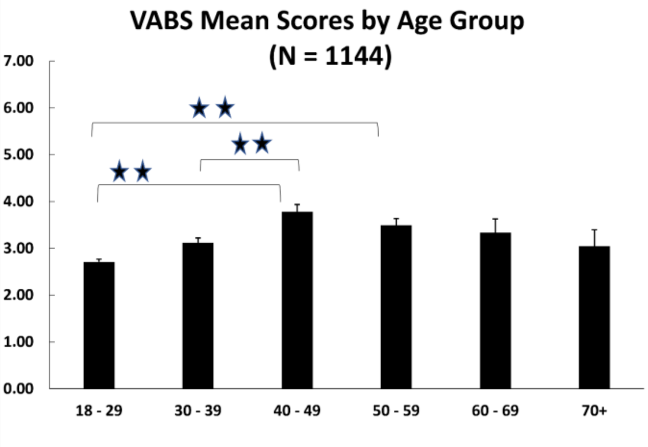

Gender Groups

Table S6

VABS mean scores for gender groups

Gender	N	Mean	SD
Female	773	3.11	1.658
Male	361	3.06	1.599
diverse	10	2.51	1.703
Total	1144	3.09	1.640

Dependent Variable: VABS

A significant effect for gender was not observed, $F(2, 1141) = .723$, $p = .49$.

Latent Profile Analysis (LPA)

Table S7

Correlation Matrix of Vaccination Status and Measures used for LPA

Measures	1	2	3	4	5	6	7	8
1. Vaccinated (0/1)	1.000	-.669**	-.649**	.110**	-.011	-.090**	-.045	.089**
2. VABS	-.669**	1.000	.816**	-.096**	.134**	.230**	.107**	-.015
3. Conspiracy Beliefs	-.649**	.816**	1.000	-.095**	.139**	.227**	.091**	-.053*
4. Neuroticism	.110**	-.096**	-.095**	1.000	.324**	.246**	.419**	.591**
5. Powerful Others	-.011	.134**	.139**	.324**	1.000	.633**	.301**	.378**
6. Chance	-.090**	.230**	.227**	.246**	.633**	1.000	.281**	.320**
7. Hostility	-.045	.107**	.091**	.419**	.301**	.281**	1.000	.595**
8. Interpersonal Sens.	.089**	-.015	-.053*	.591**	.378**	.320**	.595**	1.000

Note. Spearman's rho, * $p < .05$, ** $p < .01$.

Mplus Input File for LPA

```
Title:          Latent Profile Analysis, 5 Classes

Data:           File = LPA.dat;

Variable:       Names =
                ! Unvacc = unvaccinated (no / yes)
                ! Neuroticism, External Control Powerful others and Chance
                ! V26 = Item: Tiny devices in vaccine
                ! V27 = Item: Genetic Guinea pig
                ! VABS14 = mean of 14 item VABS
                ! AGGR5 = BSI Aggression, 5 response categories
                ! INTSENS5 = BSI Interpersonal Sensitivity, 5 response categories
                No Unvacc Age Sex
                NEURO POWER CHANCE
                V26 V27 VABS14 AGGR5 INTSENS5;

! These variables are additionally included in the new data file
                Auxiliary = No Unvacc Age Sex;

! Variables used in this analysis
                USEVARIABLES =
                NEURO POWER CHANCE
                AGGR INTSENS VABS14 V2627;

! Label and number of classes for latent class variable
                Classes = c(5);

! Missing values are denoted by -99
          Missing = all(-99);
DEFINE:         AGGR = (AGGR5/5)*7;
                INTSENS = (INTSENS5/5)*7;
                VABS14MW = VABS14/14;
                V2627 = (V26_1 + V27_1)/2; !extreme conspiracy beliefs
                ! All variables are rescaled to 7 response categories

ANALYSIS:       TYPE = MIXTURE;   ! Requests a mixture distribution analysis (LPA)
          STARTS = 500 100;     ! Increases the number of random starts in the
                                ! 1st and 2nd step of the optimization
         STITERATIONS = 100;    ! Increases the number of iterations in the
                                ! 1st step of the optimization
         LRTBOOTSTRAP = 30;     ! Increases the number of bootstrap draws
! TECH11 = Requests the Lo-Mendell-Rubin likelihood ratio test of model fit
! TECH14 = LR (k-1) difference test; SVALUES = Output of starting values
OUTPUT:         TECH11; TECH14; SVALUES;

PLOT:           TYPE=PLOT3; ! Plots the estimated means in each class
                     SERIES IS       ! Order of variables that are shown on the x-axis
                     NEURO(1) CHANCE(2) POWER(3) AGGR(4) INTSENS(5)
                     VABS14(6) V2627(7);
```

! Saves individual class assignment prob. and estimated class membership to an external data file
SAVEDATA: FILE = LPA_5classes.dat;
SAVE = CPROBABILITIES;

We varied and increased the random starts to avoid local solutions. These are the final stage loglikelihood (LL) values at local maxima, seeds, and initial stage start numbers.

Table S8

LPA output: loglikelihood (LL) values

LL at Local Maxima	Seeds	Initial Stage Start Numbers
-9305.982	25127	107
-9305.982	535804	111
-9305.982	937885	426
-9305.982	937225	394
-9305.982	605565	404
-9305.982	61587	400
-9305.982	714997	399
…	...	…
-9305.982	507154	387
-9305.982	81117	305
-9379.160	629320	222

THE BEST LOGLIKELIHOOD VALUE HAS BEEN REPLICATED. RERUN WITH AT LEAST TWICE THE RANDOM STARTS TO CHECK THAT THE BEST LOGLIKELIHOOD IS STILL OBTAINED AND REPLICATED.

Table S9

Average latent class probabilities for most likely latent class membership (row) by latent class (column)

Latent Classes	Class Counts	Proportions
1	711	0.611
2	122	0.119
3	189	0.162
4	40	0.037
5	82	0.070

Table S10

Final class counts and proportions for the latent classes based on their most likely class membership

	Class1	Class2	Class3	Class4	Class5
1	**0.938**	0.000	0.000	0.045	0.017
2	0.000	**0.938**	0.062	0.000	0.000
3	0.000	0.030	**0.967**	0.003	0.000
4	0.010	0.004	0.022	**0.944**	0.020
5	0.042	0.001	0.000	0.016	**0.941**

Table S11

Distribution of mental health problems across latent classes

	Class 1<br>*N* (%)	Class 2<br>*N* (%)	Class 3<br>*N* (%)	Class 4<br>*N* (%)	Class 5<br>*N* (%)	Total<br>*N* (%)
Affirmed diagnosis	94 (13.2)	74 (60.7)	28 (14.8)	13 (32.5)	8 (9.8)	217 (100)
Specific diagnoses						
Depression	34 (36.2)	2 (2.7)	--	--	--	36 (38.9)
Anxiety	12 (12.8)	2 (2.7)	--	--	--	14 (15.5)
PTBS	5 (5.3)	--	--	--	--	
Multimorbid	30 (31.9)	2 (2.7)	--	--	--	32 (34.6)
Other	7 (7.4)	2 (2.7)	--	--	--	9 (10.1)
Did not specify	6 (6.4)	66 (89.2)	28 (100)	13 (100)	8 (100)	121 (55.8)

Table S12

Significance Tests for Class Comparisons per Construct.

Comparison of Classes	Estimate	SE	Est/SE	Two-Tailed P-Value
**Neuroticism**				
1-2	-1.805	0.107	-16.919	0.000
1-3	0.070	0.132	0.529	0.597
1-4	-1.163	0.215	-5.405	0.000
1-5	0.421	0.197	2.141	0.032
2-3	1.875	0.148	12.684	0.000
2-4	0.642	0.225	2.847	0.004
2-5	2.226	0.210	10.620	0.000
3-4	-1.233	0.230	-5.349	0.000
3-5	0.351	0.229	1.536	0.124
4-5	1.584	0.271	5.853	0.000
**Powerful Others**				
1-2	-0.841	0.078	-10.815	0.000
1-3	-0.199	0.073	-2.736	0.006
1-4	-1.106	0.170	-6.497	0.000
1-5	-0.371	0.146	-2.544	0.011
2-3	0.642	0.103	6.220	0.000
2-4	-0.265	0.187	-1.419	0.156
2-5	0.470	0.164	2.871	0.004
3-4	-0.907	0.178	-5.105	0.000
3-5	-0.172	0.163	-1.055	0.291
4-5	0.735	0.219	3.357	0.001
**Chance**				
1-2	-0.810	0.088	-9.234	0.000
1-3	-0.366	0.071	-5.127	0.000
1-4	-1.169	0.166	-7.040	0.000
1-5	-0.601	0.136	-4.428	0.000
2-3	0.443	0.110	4.018	0.000
2-4	-0.359	0.186	-1.929	0.054
2-5	0.209	0.160	1.302	0.193
3-4	-0.802	0.173	-4.631	0.000
3-5	-0.235	0.152	-1.544	0.123
4-5	0.568	0.208	2.729	0.006
**Hostility**				
1-2	-1.369	0.188	-7.297	0.000
1-3	-0.163	0.054	-3.019	0.003
1-4	-2.426	0.200	-12.140	0.000
1-5	-0.115	0.076	-1.514	0.130
2-3	1.207	0.205	5.875	0.000
2-4	-1.057	0.271	-3.896	0.000
2-5	1.254	0.211	5.935	0.000
3-4	-2.263	0.204	-11.107	0.000
3-5	0.048	0.089	0.535	0.593
4-5	2.311	0.205	11.288	0.000

Table S12 cont'd

Comparison of Classes	Estimate	SE	Est/SE	Two-Tailed P-Value
**Interpersonal Sensitivity**				
1-2	-2.202	0.170	-12.921	0.000
1-3	-0.025	0.070	-0.348	0.728
1-4	-2.406	0.243	-9.899	0.000
1-5	0.097	0.088	1.110	0.267
2-3	2.178	0.204	10.673	0.000
2-4	-0.204	0.312	-0.653	0.513
2-5	2.300	0.212	10.841	0.000
3-4	-2.382	0.241	-9.864	0.000
3-5	0.122	0.103	1.180	0.238
4-5	2.503	0.244	10.263	0.000
**VABS**				
1-2	-0.107	0.098	-1.099	0.272
1-3	-2.805	0.086	-32.697	0.000
1-4	-3.154	0.171	-18.473	0.000
1-5	-3.875	0.116	-33.490	0.000
2-3	-2.698	0.120	-22.407	0.000
2-4	-3.046	0.189	-16.128	0.000
2-5	-3.768	0.143	-26.430	0.000
3-4	-0.348	0.188	-1.849	0.064
3-5	-1.070	0.122	-8.790	0.000
4-5	-0.721	0.201	-3.596	0.000
**Extreme Conspiracy Beliefs**				
1-2	0.008	0.029	0.278	0.781
1-3	-2.134	0.083	-25.627	0.000
1-4	-3.264	0.168	-19.413	0.000
1-5	-4.304	0.134	-32.048	0.000
2-3	-2.142	0.088	-24.306	0.000
2-4	-3.272	0.170	-19.292	0.000
2-5	-4.312	0.137	-31.461	0.000
3-4	-1.130	0.175	-6.461	0.000
3-5	-2.170	0.110	-19.718	0.000
4-5	-1.040	0.197	-5.286	0.000

Latent Class Membership by Age Group

Figure S2

Percentages of class membership for each age group

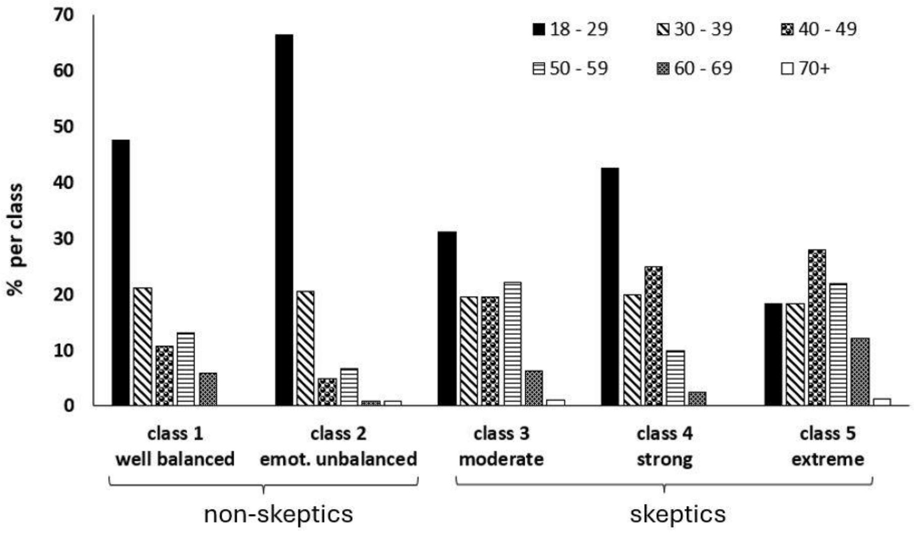

Latent Class Membership by Gender Group

Figure S3

Percentages of class membership for each gender group

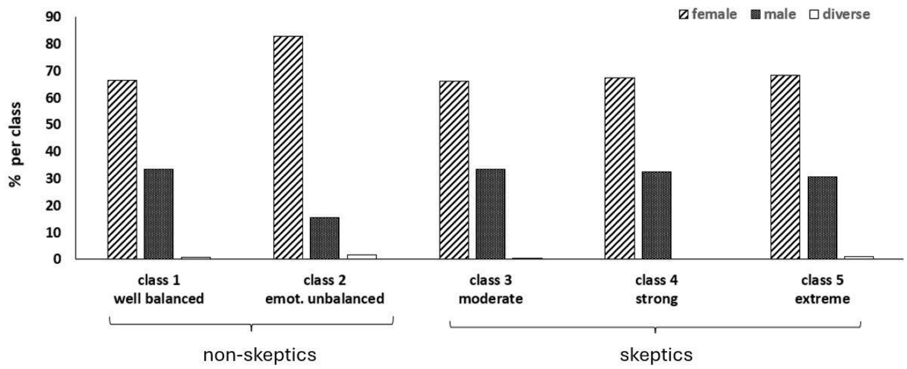

Level of Education by Latent Class Membership

Figure S4

Percentage of level of education by latent class membership

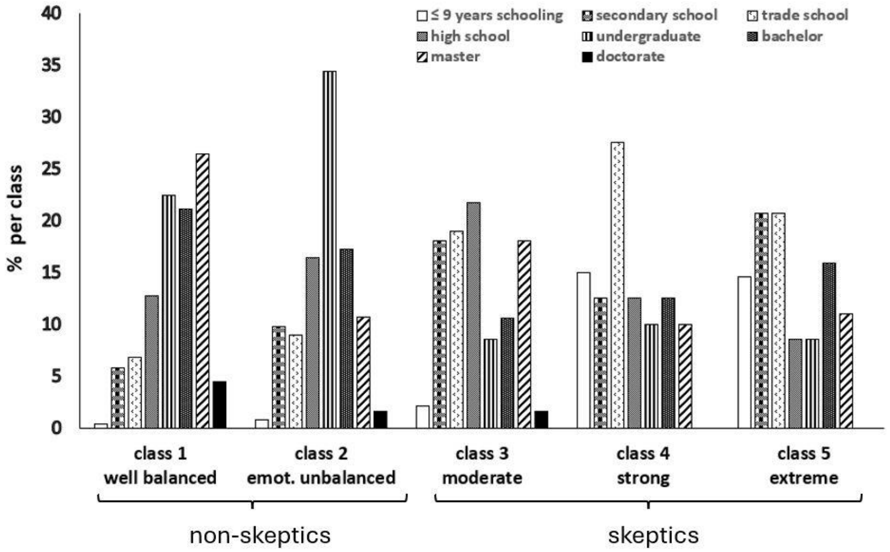

Supplement: Supplemental Material - Alike but not the same: Psychological profiles of COVID-19 vaccine skeptics [file sj-pdf-3-hpo-10.1177_20551029241248757.pdf]
